# Allelopathy and potential allelochemicals of *Ligularia sagitta* as an invasive plant

**DOI:** 10.1080/15592324.2024.2335025

**Published:** 2024-04-28

**Authors:** Shengxiao Wang, Chenyue Wang, Jun Zhang, Kan Jiang, Fang Nian

**Affiliations:** a College of Science, Gansu Agricultural University, Lanzhou, China; b College of Agronomy, Gansu Agricultural University, Lanzhou, China

**Keywords:** *Ligularia sagitta*, allelopathy, inhibition, allelochemical, forage

## Abstract

Allelopathy is the main chemical means in the invasion process of exotic plants and one of the key factors in grassland degradation. In this experiment, we investigated the effects of ethyl acetate phase extract (EAE), n-butanol phase extract (BE) and aqueous phase extract (AE) from the aboveground (stems and leaves) and roots of *Ligularia sagitta* on seed germination and seedling growth of four *Gramineae* forages (*Poa pratensis* L. *Festuca ovina* L. *Elymus nutans* Griseb. *Agropyron cristatum* (L.) Gaertn.) in their sympatric domains and one *Legosuminae* forage (*Medicago sativa* L.). The chemical components in each phase extract of *L. sagitta* were determined with UHPLC-MS/MS non-targeted metabolomics, and the differential compounds were screened using Orthogonal Partial Least Squares-Discriminant Analysis (OPLS-DA). Within a set concentration range, EAE significantly inhibited seed germination and seedling growth of four *Gramineae* forages. BE and AE acted mainly in the seedling growth stage and did not significantly inhibit forage seed germination. *P. pratensis* was most sensitive to *L. sagitta* extracts; at 2.0 mg/mL of EAE from roots, germination energy and germination rate of *P. pratensis* seeds were 0. *L. sagitta* extracts inhibited the growth of *M. sativa* seedlings and did not inhibit its seed germination. A total of 904 compounds were identified with UHPLC-MS/MS, among which 31, 64, 81 and 66 metabolites displayed different accumulation patterns in the four comparison groups (R.EAE vs. R.BE, R.EAE vs. R.AE, SL.EAE vs. SL.BE, SL.EAE vs. SL.AE), respectively. In particular, 9 compounds were found to be common up-regulated differential metabolites in the four comparison groups and were enriched in EAE. Additionally, N,N-dimethylaniline, Caffeic acid, 4-Hydroxybenzoic acid, 4-Hydroxybenzaldehyde and cis-9-Octadecenoic acid as potential allelochemicals in *L. sagitta*. The results of this study support efforts at finding alternative control plants for the restoration of poisonous grass-type degraded grasslands.

## 1. Introduction

In the past few decades, Plant invasions have intensified globally as a result of human activities, ongoing climate change and the destruction of natural habitats.^1,2^ Although several invasive plant species bring some benefits to the invaded sites, they also have many negative impacts on local biodiversity and ecosystems.^3,4^ The ability of invasive plant species to deplete native biodiversity through competition or the secretion of secondary metabolites and to alter ecological succession in natural habitats by altering the soil microbial environment and physicochemical properties has been demonstrated in many studies.^5,6^ It has been shown that most invasive plant species invade grassland ecosystems.^7^ Grassland ecosystems are vital for the development of animal husbandry and sustainability of specific ecological functions in environmental protection and biodiversity conservation.^8^ However, grassland ecosystems are fragile and vulnerable to climate change and anthropogenic disturbance, and they can easily become new habitats for invasive alien species.^9^ An invasion of grasslands by exotic organisms leads to degradation, including a change in dominant species from native to invasive and associated reduction of good forage and decrease in biodiversity.^10^ Grassland invasions by exotic species are directly associated with livestock production losses and indirectly associated with economic losses for the prevention and control of invasive alien species.^11^

Most invasive plant species exhibit allelopathic effects, indicating that the role of allelopathy in promoting species invasions may be widespread.^12^ Plant allelopathy refers to the release of allelochemicals into the environment through root secretions and subsequent rainwater leaching, which directly or indirectly affects the germination and growth of other plants.^13^ The release of allelochemicals is a survival strategy evolved by species to obtain ecological advantages in special habitats, which may affect the growth of other plants and the succession of plant communities in the same habitat, and even affect the distribution pattern and biodiversity of plant populations in a region.^14,15^ When allelochemicals contact or approach the target plant, they directly interfere with normal plant processes such as photosynthesis and respiration, or indirectly affect the target species by changing the surrounding environment, especially soil physical, chemical and microbial properties.^16–18^

A number of studies have shown that the plant tissue and root soil extracts of poisonous exotic weeds can reduce seed germination rate and seedling biomass of tested plants,^19,20^ and that the allelopathic effects differed significantly among the sources, extract concentrations and plant species tested. In general, allelochemicals of invasive plants have little effect in their native environment on the adjacent species due to long-term mutual adaptation; however, they exert a strong allelopathic interference effect as new substances in the invaded habitat.^21^

*Ligularia sagitta* is widely distributed in alpine shrublands, riverside swamps, and meadows in western China.^22^ The roots and leaves of *L. sagitta* can be used as medicine to relieve fever, cough, and nausea.^23,24^ However, *L. sagitta* also has certain toxicity. The pyrrolizidine alkaloids contained in *L. sagitta* are the main toxic components. Ingestion by livestock can cause poisoning or even death, severely inhibiting the development of animal husbandry.^25,26^ *L. sagitta* has recently become a dominant species in natural grasslands in the Qinghai-Tibet Plateau, and is one of the poisonous invasive weeds in degraded grasslands.^27–29^ Studies have found that a variety of plants of the genus *Ligularia* such as *Ligularia virgaurea* and *Ligularia cymbulifera*, have shown allelopathic effects. The terpenoids contained in the plants of the genus are one of the sources of allelopathic activity.^30,31^

The purpose of this study was to examine the allelopathic activity of aboveground and roots extracts of *L. sagitta* on five forages, and its effects on germination and growth of forage seeds. The results provide a theoretical basis for future grassland restoration, and control of *L. sagitta*.

## 2. Materials and methods

### 2.1. Source of plant material

The whole plant of *L. sagitta* in vigorous growth period was collected from Maqu County, Gannan Tibetan Autonomous Prefecture, Gansu Province (E: 100°45′45′′-102°29′00′′, N: 33°06′30′′-34°30′15′′) in August 2021, and a total of 80 kg of fresh samples were collected. It was identified as *L. sagitta* by Professor Guo Yehong of Gansu Agricultural University. The soil on the plant was brushed clean and the plant was divided into two parts: aboveground (stems-leaves) and roots, which were air-dried and pulverized for use.

### 2.2. Production of extracts

Dried stems-leaves (7.6 kg) and roots (6.1 kg) samples of *L. sagitta* were soaked in 95% ethanol and extracted three times after 7, 5, and 3 d. The extracts obtained were combined and filtered through filter paper. The filtrate was concentrated on a rotary evaporator (40 °C) to yield the residue of stems-leaves (432 g) and roots (339 g) of *L. sagitta*. The resulting stems-leaves extracts and roots extracts were suspended in warm water (40 °C), respectively. And sequentially subjected to graded extraction with equal volumes of two solvents of different polarity, ethyl acetate and n-butanol. Each solvent extraction was repeated three times. The extracts obtained were combined and concentrated to dryness using a rotary evaporator along with the aqueous phase left after extraction to obtain the roots ethyl acetate extract (R.EAE, 189 g), stems-leaves ethyl acetate extract (SL.EAE, 216 g), roots n-butanol extract (R.BE, 56 g), stems-leaves n-butanol extract (SL.BE, 67 g), roots aqueous extract (R.AE, 35 g) and stems-leaves aqueous extract (SL.AE, 42 g). All extracts were stored in a refrigerator at 4°C.

### 2.3. Forage seeds

Seeds of four *Gramineae* forages (*Poa pratensis* L., *Festuca ovina* L., *Elymus nutans* Griseb., *Agropyron cristatum* (L.) Gaertn.) were collected in September 2021 in Maqu County, Gannan Tibetan Autonomous Prefecture, China (E: 100°45′45′′-102°29′00′′, N: 33°06′30′′-34°30′15′′). And one *Legosuminae* forage (*Medicago sativa* L.) were provided by the Hengyou Ecological Company in Lanzhou City, Gansu Province (September 2021). The four *Gramineae* species tested in this study were native to the *L. sagitta* invasion site, and *M. sativa* was a planted forage. Full and healthy seeds were selected for seed germination experiment.

### 2.4. *Allelopathic activity determination of* L. sagitta *extracts*

The allelopathic activity was determined according to a previously reported method with some modifications.^32,33^ The extracts of each phase (extracts prepared with different solvents) were accurately weighed, dissolved in a small amount of methanol and diluted with distilled water (working concentration ranges of 0.25, 0.50, 0.75, 1.0, and 2.0 mg/mL). The final concentration of methanol never exceeded 1.6%. Seeds were sterilized by immersion in 75% ethanol for 3 min, followed by several rinses with sterilized distilled water. Petri dishes (90 × 15 mm) were pre-lined with two layers of filter paper and moistened with 4 mL of the previously configured solution; the seeds were evenly placed in the Petri dishes with 30 seeds in each dish. The control group was placed in the same concentration of methanol aqueous solution as the experimental groups. Three replicates were set for each treatment.

Petri dishes were placed in an incubator for 12 days (light: 12 h 10,000 lux, 180 μmol m^−2^ s^−1^/dark: 12 h, 0 lux; 23 ± 0.5 °C). Distilled water was added regularly to maintain the extract concentration during culture. Germination energy (GE) is defined as the number of seeds germinated during the first 1/3 of the specified period of the germination test as a percentage of the number of seeds tested. Germination rate (GR) means the number of seeds germinated within a specified germination period as a percentage of the total number of seeds tested. GE was counted on the 4th day, and the GR was counted at the end of the experiment on the 12th day, and the calculations are shown in equations (1) and (2), respectively. Ten seedlings were randomly selected from each Petri dish, and root length and shoot length of the seedlings were measured with a digital Vernier caliper (0-150 mm, Harbin Measuring & Cutting Tool Group Co.,Ltd, China); fresh weight of the seedlings was determined with an analytical balance with precision to ten-thousandth of a gram. (If the number of germinated seeds was less than 10, growth parameters of all seedlings in the Petri dish were measured and the average value was calculated).

(1) $$\mathrm{GR}=\frac{\mathrm{Number}\;\mathrm{of}\;\mathrm{seeds}\;\mathrm{germinated}\;\mathrm{in}\;\mathrm{the}\;\mathrm{first}4\mathrm{days}}{\mathrm{Number}\;\mathrm{of}\;\mathrm{seeds}\;\mathrm{for}\;\mathrm{testing}}\times 100\%$$

(2) $$\mathrm{GE}=\frac{\mathrm{Number}\;\mathrm{of}\;\mathrm{seeds}\;\mathrm{germinated}\;\mathrm{within}12\mathrm{days}}{\mathrm{Number}\;\mathrm{of}\;\mathrm{seeds}\;\mathrm{for}\;\mathrm{testing}}\times 100\%$$

### 2.5. UHPLC-MS/MS analysis

Chromatographic (Vanquish UHPLC, Thermo Fisher, Germany) conditions: Hypersil Gold column (100 × 2.1 mm, 1.9 μm, Thermo Fisher, USA), Gradient elution was performed at a flow rate of 0.2 mL/min and a column temperature of 40°C. For the positive ion mode, mobile phase A was 0.1% formic acid aqueous solution and mobile phase B was methanol. For the negative ion mode, mobile phase A was 5 mM ammonium acetate solution (pH = 9.0), and mobile phase B was methanol. The gradient elution procedure was 0–1.5 min with 2% B/A, and kept for 1.5 min; 1.5–3 min with 2–85% B/A; 3–10 min with 85–100% B/A; 10–10.1 min with 100–2% B/A; 10.1–12 min with 2% B/A.

Mass spectrometry (Q ExactiveTM HF, Thermo Fisher, Germany) conditions: an electron spray ionization (ESI) instrument was used with a scanning range m/z of 100–1500; ESI was set to spray voltage of 3.5 Kv, sheath gas flow rate of 35 psi, auxiliary gas flow rate of 10 L/min, capillary temperature of 320°C, and polarity: positive, negative; MS/MS secondary scanning was data-dependent.

### 2.6. Data processing and multivariate analysis

Chemical analysis was based on secondary profiling information obtained from public databases of metabolite information mzCloud (https://www.mzcloud.org/), mzVault, and Masslist. After obtaining the spectral analysis data of metabolites in different samples, peak identification, integration, retention time correction, peak alignment, and mass spectrometry fragment attribution analysis were performed. The qualitative results and relative contents of the compounds were obtained after standardization of the data.

Multivariate statistical analysis of metabolomics data was performed using SIMCA 14.0 (Umetrics, Umeå, Sweden) software for hierarchical cluster analysis (HCA), principal component analysis (PCA), and orthogonal partial least squares discrimination analysis (OPLS-DA). OPLS-DA is a multivariate statistical analysis method with supervised pattern recognition that can effectively screen for differential metabolites by excluding study-irrelevant effects. Allelopathic data were tested for normality, homogeneity of variance and single factor analysis of variance (ANOVA) using SPSS 26.0 (Armonk, NY, USA). Correlations between the independent (extract concentration) and dependent variables (germination energy, germination rate, root length, shoot length and fresh weight) were tested separately using ANOVA and significance was set to (*p* < .05). Finally, Origin 2021 (Hampton, Massachusetts, USA) is used for graphing the figures.

## 3. Results

### 3.1. *Effects of* L. sagitta *extracts on seed germination*

Previous experiments showed that low concentration of methanol had no effect on the germination of grass seeds. Therefore, solvent interference was ignored.^32^ The results showed that BE and AE had no significant inhibitory effect on germination of the tested forages, but EAE significantly inhibited germination of the four *Gramineae* forages, and the degree of inhibition gradually increased with the increase in extract concentration. The extracts of *L. sagitta* had no effect on the germination of *M. sativa* seeds.

#### 3.1.1. Effects on germination energy of forage seeds

**Figure 1.**
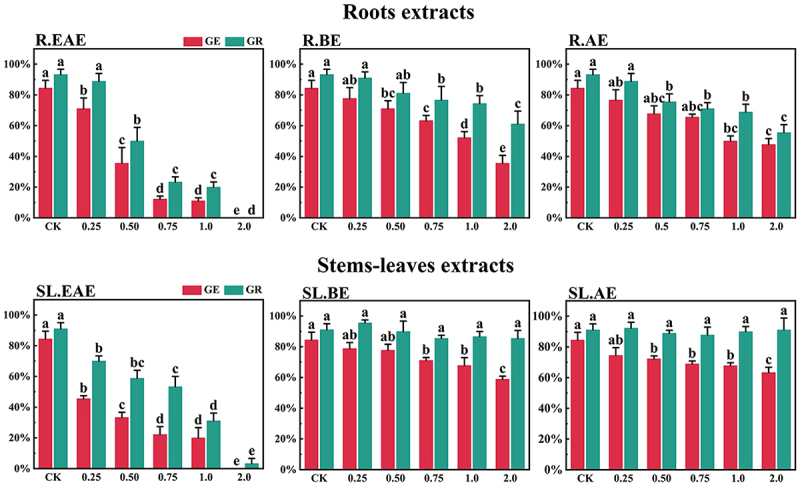
Effects of *L. sagitta* extracts on seed germination of *P. pratensis*. a: roots ethyl acetate extract (R.EAE), b: roots n-butanol extract (R.BE), c: roots aqueous extract (R.AE), d: stems-leaves ethyl acetate extract (SL.EAE), e: stems-leaves n-butanol extract (SL.BE), f: stems-leaves aqueous extract (SL.AE). In each graph, different letters indicate significant differences between concentration treatments, *p* < .05. Concentration units for treatment groups are mg/mL. Error bars indicate standard deviation (SD). (GE) germination energy; (GR) germination rate; (CK) control group.

**Figure 2.**
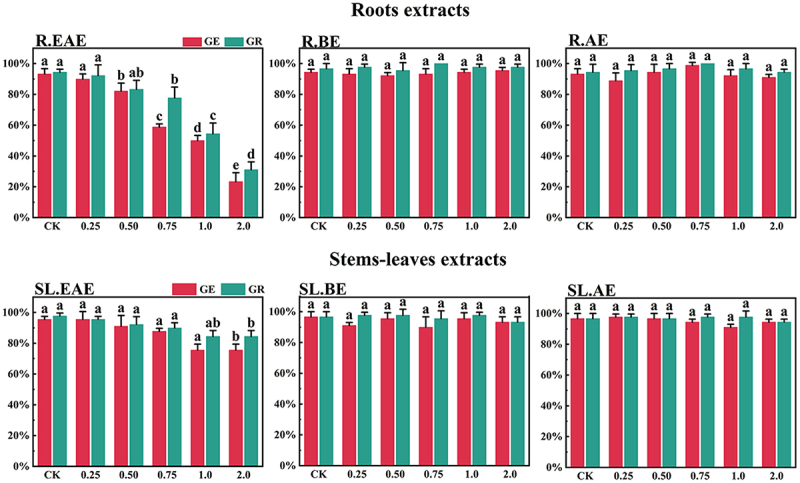
Effects of *L. sagitta* extracts on seed germination of *F. ovina*. a: roots ethyl acetate extract (R.EAE), b: roots n-butanol extract (R.BE), c: roots aqueous extract (R.AE), d: stems-leaves ethyl acetate extract (SL.EAE), e: stems-leaves n-butanol extract (SL.BE), f: stems-leaves aqueous extract (SL.AE). In each graph, different letters indicate significant differences between concentration treatments, *p* < .05. Concentration units for treatment groups are mg/mL. Error bars indicate standard deviation (SD). (GE) germination energy; (GR) germination rate; (CK) control group.

**Figure 3.**
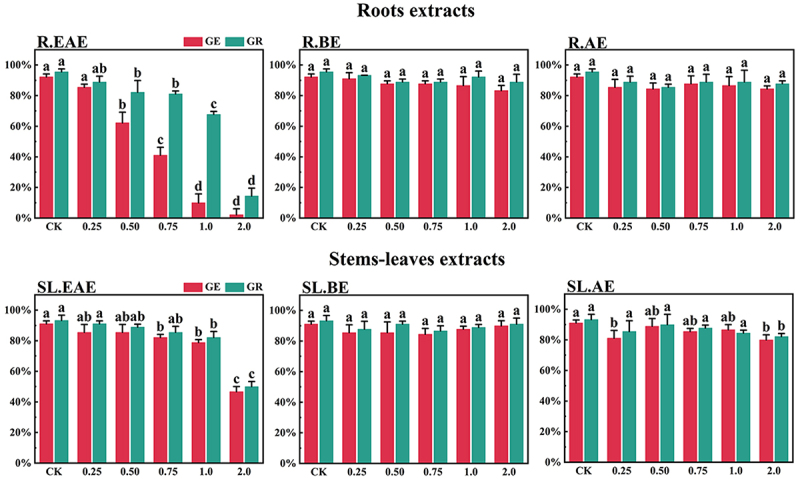
Effect of *L. sagitta* extracts on seed germination of *E. nutans*. a: roots ethyl acetate extract (R.EAE), b: roots n-butanol extract (R.BE), c: roots aqueous extract (R.AE), d: stems-leaves ethyl acetate extract (SL.EAE), e: stems-leaves n-butanol extract (SL.BE), f: stems-leaves aqueous extract (SL.AE). In each graph, different letters indicate significant differences between concentration treatments, *p* < .05. Concentration units for treatment groups are mg/mL. Error bars indicate standard deviation (SD). (GE) germination energy; (GR) germination rate; (CK) control group.

**Figure 4.**
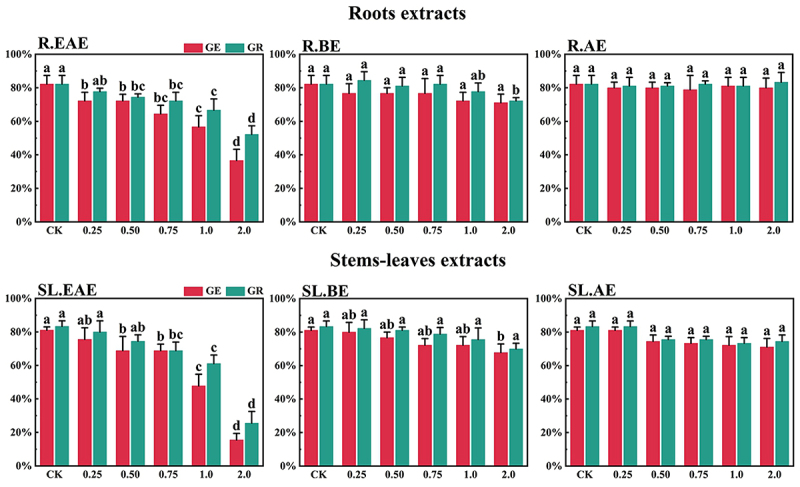
Effect of *L. sagitta* extracts on seed germination of *A. cristatum*. a: roots ethyl acetate extract (R.EAE), b: roots n-butanol extract (R.BE), c: roots aqueous extract (R.AE), d: stems-leaves ethyl acetate extract (SL.EAE), e: stems-leaves n-butanol extract (SL.BE), f: stems-leaves aqueous extract (SL.AE). In each graph, different letters indicate significant differences between concentration treatments, *p* < .05. Concentration units for treatment groups are mg/mL. Error bars indicate standard deviation (SD). (GE) germination energy; (GR) germination rate; (CK) control group.

**Figure 5.**
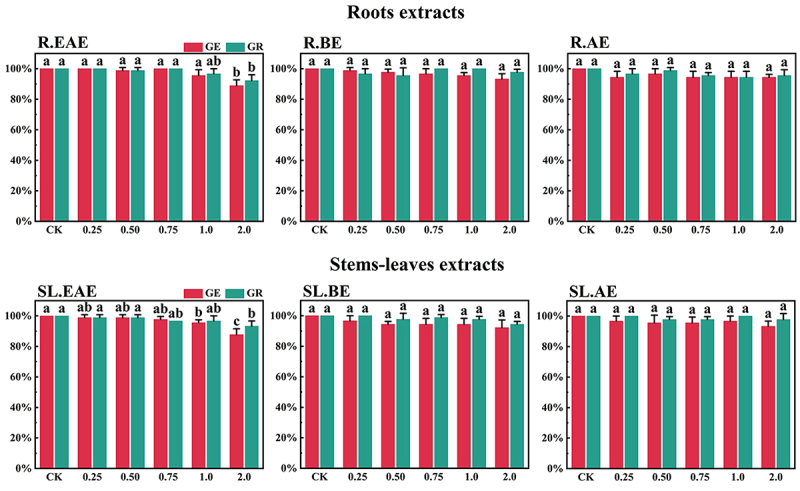
Effects of *L. sagitta* extracts on seed germination of *M. sativa*. a: roots ethyl acetate extract (R.EAE), b: roots n-butanol extract (R.BE), c: roots aqueous extract (R.AE), d: stems-leaves ethyl acetate extract (SL.EAE), e: stems-leaves n-butanol extract (SL.BE), f: stems-leaves aqueous extract (SL.AE). In each graph, different letters indicate significant differences between concentration treatments, *p* < .05. Concentration units for treatment groups are mg/mL. Error bars indicate standard deviation (SD). (GE) germination energy; (GR) germination rate; (CK) control group.

*L*. *sagitta* extracts significantly inhibited germination energy (GE) of *P. pratensis* seeds and delayed germination of *P. pratensis* seeds (Figure 1). At 2.0 mg/mL of EAE, germination energy of *P. pratensis* seeds decreased to 0, and germination energy of *E. nutans*, *A. cristatum*, and *F. ovina* seeds decreased to 2.2, 15.6, and 23.3%, respectively (Figures 3, Figures 4, 2). The inhibitory effect of R.EAE on germination energy of *F. ovina* and *E. nutans* was greater than that of SL.EAE, but that on *A. cristatum* was less than that of SL.EAE. The extracts of *L. sagitta* had no significant effect on germination energy of *M. sativa* seeds (Figure 5).

#### 3.1.2. Effects on germination rate of forage seeds

The inhibition effect of *L. sagitta* extracts on the germination rate (GR) of four *Gramineae* forage seeds was weaker than that on the germination energy. At 0.75 mg/mL of R.EAE, the germination rate of *P. pratensis* seeds was 23.3% and it decreased to 0 at 2.0 mg/mL of R.EAE (Figure 1). Seed germination of *E. nutans* and *F. ovina* exhibited similar results, and the inhibition effect was greatest at 2.0 mg/mL of R.EAE. The seed germination rate for *E. nutans* was 14.4% (Figure 3), and that for *F. ovina* was 31.1% (Figure 2), which was significantly different from the control. The effect of stems-leaves extracts on seed germination of *F. ovina* was not significant. For *A. cristatum*, the inhibitory effect of stems-leaves extracts was greater than that of roots extracts (Figure 4). Germination rate of *M. sativa* seeds was above 90.0%, which was not significantly different from that of the control, and its germination was not affected by *L. sagitta* extract (Figure 5).

### 3.2. *Effects of* L. sagitta *extracts on the growth of forage seedlings*

EAE had the most significant inhibitory effect on shoot length of forage grass seedlings, and severely inhibited shoot length of four *Gramineae* species at concentrations >0.50 mg/mL; at the highest concentration (2.0 mg/mL), the inhibition of shoot length of *P. pratensis* was 87.7% (Table 1), and that of *E. nutans* was 87.0% (Table S2). BE and AE significantly inhibited shoot growth of *P. pratensis* seedlings, but had no significant inhibitory effect on shoot length of *E. nutans* seedlings. SL.BE and SL.AE did not significantly inhibit shoot length of *F. ovina* or *A. cristatum*. All extracts inhibited shoot length of *M. sativa* seedlings to the same extent, and the degree of inhibition increased with increasing extract concentration. Overall, roots extracts inhibited shoot length more than stems-leaves extracts.

**Table 1.** Effects of *L. sagitta* extracts on *P. pratensis* seedling growth.

Treatment solution		Inhibition（%）					
		Roots extracts			Stems-Leaves extracts		
		Shoots	Roots	Fresh weight	Shoots	Roots	Fresh weight
EAE (mg/mL)	0.25	16.47 ± 4.55 g	41.18 ± 0.52 g	16.87 ± 1.92 ef	18.59 ± 2.87 e	39.68 ± 1.11 f	25.76 ± 1.98 def
	0.50	44.41 ± 1.52 c	61.85 ± 0.71 b	36.15 ± 4.87 cd	47.52 ± 3.27 d	59.48 ± 2.90 e	30.09 ± 3.00 de
	0.75	62.49 ± 4.50 b	83.01 ± 2.05 c	50.12 ± 1.91 b	62.93 ± 4.29 c	76.83 ± 0.76 c	46.32 ± 4.35 c
	1.00	72.26 ± 3.19 a	89.96 ± 0.53 b	64.58 ± 1.91 a	72.63 ± 3.74 b	89.24 ± 0.46 b	59.52 ± 2.46 b
	2.00	——	——	——	87.65 ± 0.77 a	100.00 ± 0.00 a	72.08 ± 1.72 a
BE (mg/mL)	0.25	19.70 ± 2.92 fg	45.2 ± 1.07 f	11.33 ± 2.23 f	2.92 ± 2.00 f	33.10 ± 0.77 fg	9.96 ± 3.12 f
	0.50	22.46 ± 2.82 fg	45.88 ± 1.39 f	14.94 ± 3.56 ef	6.57 ± 3.54 f	36.64 ± 1.95 fg	13.64 ± 3.44 ef
	0.75	25.54 ± 1.45 fe	50.51 ± 3.39 e	19.52 ± 4.23 e	16.45 ± 2.37 f	58.51 ± 1.64 e	15.80 ± 2.93 ef
	1.00	30.15 ± 2.09 de	61.48 ± 0.93 d	34.94 ± 2.61 d	16.46 ± 2.37 e	58.79 ± 1.40 e	22.29 ± 4.70 def
	2.00	34.47 ± 1.24 d	83.70 ± 0.99 c	53.49 ± 5.47 b	18.87 ± 3.66 e	69.74 ± 3.28 cd	27.49 ± 3.25 de
AE (mg/mL)	0.25	8.74 ± 2.01 h	40.97 ± 1.20 g	20.00 ± 1.67 e	1.98 ± 1.30 f	27.63 ± 4.15 gh	9.31 ± 2.19 f
	0.50	21.93 ± 3.61 fg	62.59 ± 1.20 d	22.41 ± 3.52 e	20.21 ± 3.23 e	20.70 ± 4.43 h	12.34 ± 3.95 ef
	0.75	26.00 ± 4.25 ef	80.51 ± 0.08 c	31.33 ± 2.01 d	21.54 ± 3.25 e	63.47 ± 2.96 de	18.83 ± 3.90 ef
	1.00	45.74 ± 2.68 c	88.37 ± 0.43 b	35.42 ± 3.01 cd	20.93 ± 3.09 e	65.70 ± 2.52 de	29.65 ± 3.21 de
	2.00	46.37 ± 1.49 c	100.00 ± 0.00 a	42.89 ± 2.61 c	21.55 ± 3.07 e	69.87 ± 3.46 cd	37.66 ± 3.77 cd

Values in the table are average value ± standard deviation (SD), lower case letters in the same column indicate significant differences between treatments at *p* < .05; ——indicates that the germination rate is 0. Ethyl acetate extract (EAE), n-butanol extract (BE), aqueous extract (AE).

Extract treatments significantly reduced root length of five forage seedlings, and root length inhibition rate gradually increased with the increase in extract concentration. At the highest concentration of each phase of the extract (2.0 mg/mL), root length inhibition of *P. pratensis* seedlings ranged from 69.7 to 100.0% (Table 1). *F. ovina* seedlings exhibited the maximum inhibition rate of 87.2% in the SL.EAE treatment, followed by R.AE with 73.1% (Table S1). EAE had the greatest inhibitory effect on root length of *E. nutans* and *A. cristatum*. (S.Table S2, S.Table S3). Results for *M. sativa* differed from those for the other forages, with the highest inhibition of root length ranging from 63.0 to 84.0 at 2.0 mg/mL of AE (S.Table S4) and roots turning brown in color, and with twisted and distorted morphology after the AE treatment.

Different extracts of *L. sagitta* varied in the degree of inhibition of fresh weight of the five forages. Similarly, EAE inhibited fresh weight of the forages most, with the greatest inhibition of *P. pratensis* seedlings. At 2.0 mg/mL, inhibition of fresh weight of *A. cristatum* seedlings ranged from 65.6 to 72.1% (Table 1), followed by *E. nutans* with inhibition ranging from 66.5 to 66.7%, and lowest inhibition of *M. sativa*. In addition, R.BE and R.AE significantly inhibited fresh weight of *P. pratensis* and *F. ovina* seedlings.

### 3.3. *Overall analysis of UPLC-MS/MS results of* L. sagitta *extracts*

A total of 904 components were identified from *L. sagitta* stems-leaves and roots extracts, including 154 organic acids, 102 lipids, 102 alkaloids, 86 terpenoids, 82 amino acids and their derivatives, 63 phenols, 63 heterocyclic rings, 56 carbohydrates, 41 flavonoids, 30 esters, 29 nucleotides and their derivatives, 20 coumarins, 14 lignans, 14 quinones, 14 aldehydes, and 34 kinds of other compounds.

#### 3.3.1. PCA and HCA analysis

**Figure 6.**
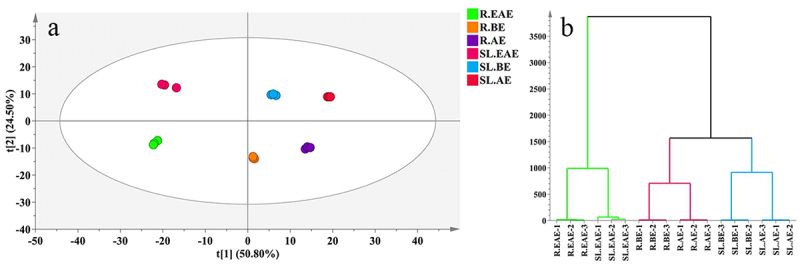
A, PCA score plot. b, HCA. Three biological repeat sequences were set up for each extraction phase (indicated by the numbers 1,2,3 at the end of the group name). Roots ethyl acetate extract (R.EAE), roots n-butanol extract (R.BE), roots aqueous extract (R.AE), stems-leaves ethyl acetate extract (SL.EAE), stems-leaves n-butanol extract (SL.BE), stems-leaves aqueous extract (SL.AE).

To gain a preliminary understanding of the overall metabolite differences between the samples in each group (extracts prepared with different solvents) and the magnitude of variability between the samples within the groups, the samples were subjected to principal component analysis (PCA) and hierarchical cluster analysis (HCA). The first two principal components in the PCA (Figure 6a) elaborated about 75.3% of total variance (PC1 = 50.8%, PC2 = 24.5%, respectively). The samples within each group were gathered together, and the separation trend between groups was obvious, indicating that there were significant differences in metabolites between stems-leaves and roots and different extraction phases of *L. sagitta*. HCA (Figure 6b) showed that the six extract phases were clustered into two major groups, and SL.EAE and R.EAE were clustered into one group, which was different from BE and AE, while the metabolite types and contents of BE and AE were closer and clustered into another group. The combined results of PCA and HCA indicate that the extracts of different parts and different extraction phases of *L. sagitta* were significantly different.

#### 3.3.2. OPLS-DA analysis

**Figure 7.**
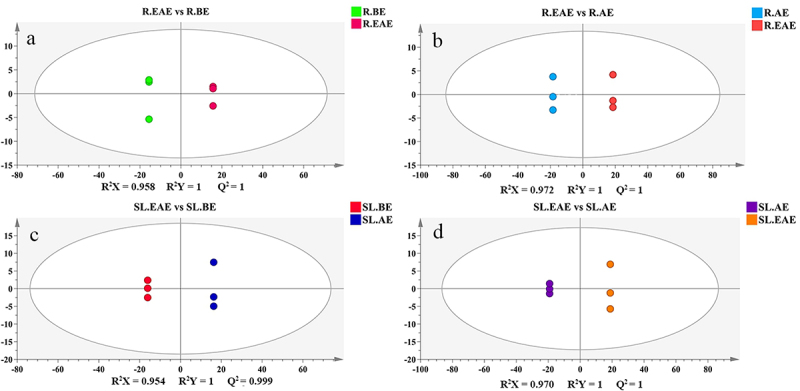
OPLS-DA score scatter plots. a: R.EAE vs. R.BE; b: R.EAE vs. R.AE; c: SL.EAE vs. SL.BE; d: SL.EAE vs. SL.AE. Roots ethyl acetate extract (R.EAE), roots n-butanol extract (R.BE), roots aqueous extract (R.AE), stems-leaves ethyl acetate extract (SL.EAE), stems-leaves n-butanol extract (SL.BE), stems-leaves aqueous extract (SL.AE). Three biological repeat sequences were set up for each extraction phase.

The results of the allelopathy tests of the extracts in subsections 3.1 and 3.2 showed that the strongest allelopathic activity in the extract of *L. sagitta* was EAE. Therefore, by comparing the differences between EAE, and BE, and EAE and AE, potential allelochemicals of *L. sagitta* were screened. Pairwise comparative was analyzed using OPLS-DA models for the different phases of stems-leaves and roots extracts (R.EAE vs. R.BE; R.EAE vs. R.AE; SL.EAE vs. SL.BE; SL.EAE vs. SL.AE). The OPLS-DA score scatter plots for each group were depicted in Figure 7a–d. To validate the OPLS-DA model, 200 permutation tests were performed in pairwise comparison groups(Fig. S1). The predictability (Q^2^) and goodness-of-fit (R^2^X, R^2^Y) exceeded 0.9 in all pairwise comparisons (R.EAE vs. R.BE, R^2^X = 0.958, R^2^Y = 1, Q^2^ = 1; R.EAE vs. R.AE, R^2^X = 0.972, R^2^Y = 1, Q^2^ = 1; SL.EAE vs. SL.BE, R^2^X = 0.954, R^2^Y = 1, Q^2^ = 0.999; SL.EAE vs. SL.AE, R^2^X = 0.970, R^2^Y = 1, Q^2^ = 1), indicating that the OPLS-DA model was sufficiently predictable and was not overfitted (Figure 7a–d). Therefore, it can be used for further screening of potential allelochemicals.The OPLS-DA score scatter plots also showed that the different extraction phases indicated a clear trend toward segregation, which proved that the compounds contained in different extraction phases were quite different.

#### 3.3.3. Screening of differential metabolites in different extraction phases

**Figure 8.**
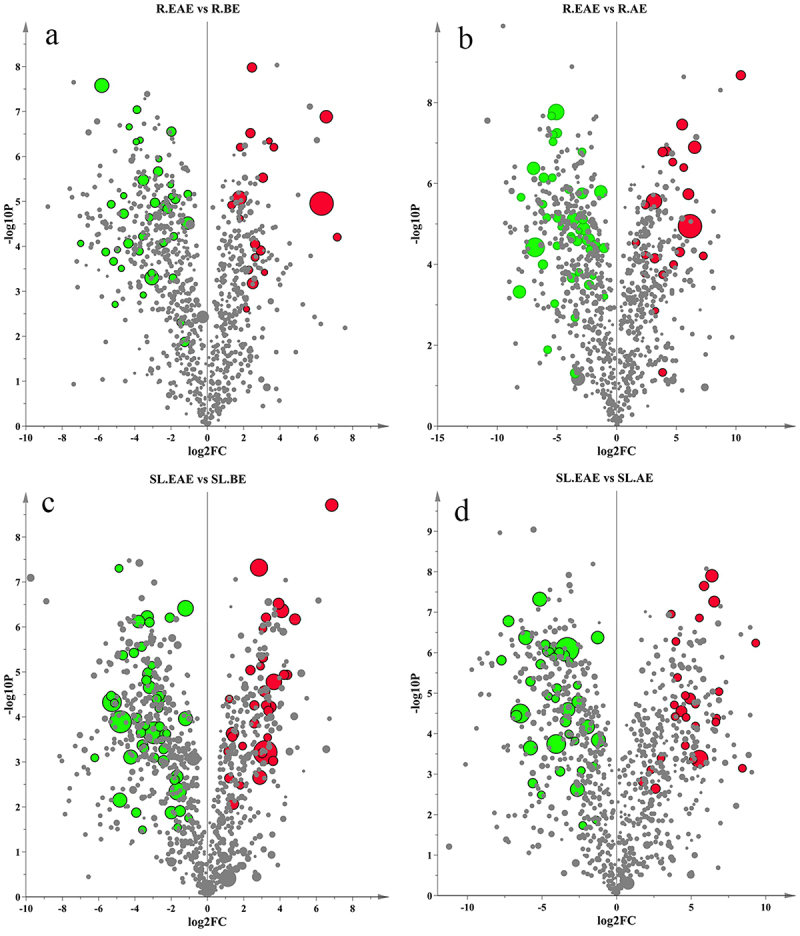
Volcano map of differential metabolites. a: R.EAE vs. R.BE; b: R.EAE vs. R.AE; c: SL.EAE vs. SL.BE; d: SL.EAE vs. SL.AE. Roots ethyl acetate extract (R.EAE), roots n-butanol extract (R.BE), roots aqueous extract (R.AE), stems-leaves ethyl acetate extract (SL.EAE), stems-leaves n-butanol extract (SL.BE), stems-leaves aqueous extract (SL.AE). Red, green and gray points indicate up-regulated, down-regulated and non-significant differential metabolites, respectively. Symbol size indicates VIP-value.

To gain more insight into the differences between R.EAE and R.BE, R.EAE and R.AE, and SL.EAE and SL.BE, and SL.EAE and SL.AE, respectively. Significantly different metabolites were screened by VIP value, *p* value, and FC (Fold Change) value. *p* value < .05, FC value ≥ 2 or ≤ 0.5 among the metabolites with a VIP value ≥ 1.5 were used as an identification criterion. A volcano diagram (Figure 8a–d) was used to show the screening results. Differential metabolites of different extracts were shown in Supplementary Table S6–9. There were 31 differential metabolites (19 up-regulated and 12 down-regulated) between R.EAE and R.BE (Figure 8a, Table S5, Table S6), 64 (21 up-regulated and 43 down-regulated) between R.EAE and R.AE (Figure 8b, Table S5, Table S7), 81 (31 up-regulated and 50 down-regulated) between SL.EAE and SL.BE (Figure 8c, Table S5, Table 8), and 66 (27 up-regulated and 39 down-regulated) between SL.EAE and SL.AE (Figure 8d, Table S5, Table S9). Attention worthy, the up-regulated differential metabolites of different extracts of *L. sagitta* roots were mainly terpenoids, lipids, alkaloids, and organic acids, whereas the up-regulated differential metabolites of different extracts of stems-leaves were mainly terpenoids, organic acids, and flavonoids.

**Figure 9.**
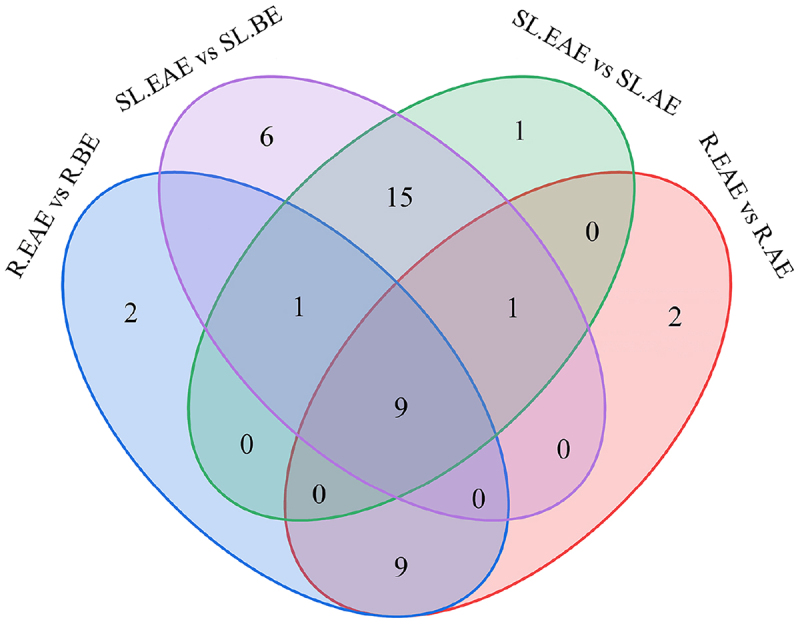
Venn diagram showing the number of overlapping and specific differential metabolites in the four comparison groups (R.EAE vs. R.BE, R.EAE vs. R.AE, SL.EAE vs. SL.BE, SL.EAE vs. SL.AE). Roots ethyl acetate extract (R.EAE), roots n-butanol extract (R.BE), roots aqueous extract (R.AE), stems-leaves ethyl acetate extract (SL.EAE), stems-leaves n-butanol extract (SL.BE), stems-leaves aqueous extract (SL.AE).

Analysis of the up-regulated compounds in the above four sets of comparisons using Venn diagrams (Figure 9) showed that nine compounds were up-regulated differential metabolites common to R.EAE vs. R.BE, R.EAE vs. R.AE, SL.EAE vs. SL.BE, and SL.EAE vs. SL.AE, which were N,N-Dimethylaniline, Caffeic acid, 2-Phenylethylamine, 4-Hydroxybenzoic acid, Eupatilin, 4-Hydroxybenzaldehyde, cis-9-Octadecenoic, 1-Monolinolein, and Schizandrol A. Their relative contents in different extracts were shown in Table 2.

**Table 2.** Relative content of 9 common differential metabolites.

Compounds	Relative content (×10^9^)					
	R.EAE	R.BE	R.AE	SL.EAE	SL.BE	SL.AE
N,N-Dimethylaniline	116.72	1.47	1.67	47.56	5.25	1.01
Caffeic acid	54.65	16.00	6.16	14.23	1.90	4.23
2-Phenylethylamine	23.13	0.25	0.25	5.55	0.68	0.16
4-Hydroxybenzoic acid	18.78	3.28	0.42	13.13	5.04	0.66
Eupatilin	13.35	2.42	0.01	5.37	0.19	0.01
4-Hydroxybenzaldehyde	11.74	2.28	0.64	5.79	1.13	0.35
cis-9-Octadecenoic acid	9.97	2.82	0.70	3.99	0.43	0.25
Schizandrol A	7.10	0.56	0.14	4.11	0.22	0.04
1-Monolinolein	4.27	0.41	0.16	7.04	0.63	0.07

Roots ethyl acetate extract (R.EAE), Roots n-butanol extract (R.BE), Roots aqueous extract (R.AE), Stems-leaves ethyl acetate extract (SL.EAE), Stems-leaves n-butanol extract (SL.BE), Stems-leaves aqueous extract (SL.AE).

## 4. Discussion

A variety of plants invade grasslands through allelopathy mechanisms, in which allelochemicals confer a competitive advantage to the invasive plant.^34^ Toxic invasive plants can reduce density of the native plant community by decreasing seed germination rate, and reducing access to light, water, and nutrients by inhibiting shoot or root growth in seedlings; this, in turn, affects the distribution pattern and biodiversity of plant populations in the region.^35–37^

Our experimental results showed that EAE of *L. sagitta* had a significant inhibitory effect on germination energy, germination rate, shoot length, root length and fresh weight of the four *Gramineae* forages, and the inhibitory effect showed concentration dependence. Germination energy reflects the homogeneity and vitality of seed germination, and the seed germination rate indicates the amount of seed germination. On the one hand, *L. sagitta* reduced the germination energy and germination rate of seeds of four *Gramineae* species and delayed seed germination, which may reduce the abundance of native species in the region^38^; On the other hand, *L. sagitta* also inhibits the root growth of forage seedlings, diminishing nutrient uptake and utilization and depriving forages of early competitiveness.^39^ This directly affects the status and role of native species in plant communities. The five tested forages showed different sensitivities to the *L. sagitta* extracts (*P. pratensis* was the most sensitive and *M. sativa* was the least sensitive to it), which may be due to the evolutionary differences in the resistance of the test forages to the allelochemicals.^15^ The strongest inhibitory effect on seed germination and seedling growth of forage was exerted by EAE, indicating that the allelochemicals of *L. sagitta* are mainly present in EAE. AE and BE had an inhibitory effect on seedling growth only, indicating that allelochemicals related to seedling growth were also present in AE and BE. It has been shown that leaves of plants with allelopathy have greater allelopathic activity than roots, but different results were shown in this study, for example, the inhibitory effect of EAE in roots of *L. sagitta* on seed germination of *P. pratensis*, *F. ovina*, *E. nutans* was greater than that of EAE in stems-leaves. This also confirms the significant differences in plant allelopathic effects among the extract sources, concentrations and plant species tested. Compared with previous reports,^20,37,40^ EAE of *L. sagitta* significantly inhibited seed germination and seedling growth of four *Gramineae* forage species at very low concentrations.

The present study also revealed that the inhibition by *L. sagitta* extracts of root length in forage seedlings was much greater than that of shoot length, which may be due to different sensitivity of different plant tissues to allelochemicals.^41^ Research has shown that rainwater leaching is one of the pathways by which plants release allelochemicals into the environment. Rainwater infiltrates the soil, and the root system becomes directly exposed to allelochemicals; therefore, the root system of most plant seedlings is usually more sensitive to external allelopathic than to other indicators.^42,43^ The above-ground parts rely on the roots to absorb nutrients to satisfy plant growth needs, therefore, only when the roots are stressed to a certain extent, water and nutrients can not be supplied normally, the other parts of the plant show symptoms of damage.^44^ We conclude that *L. sagitta* affects the growth and development of forage mainly by inhibiting the growth of roots of the forage seedlings.

The occurrence of allelopathy can be attributed to a particular compound or class of compounds.^34^ Allelochemicals are found in large quantities in the secondary metabolites of plants and are mainly categorized into phenols, terpenoids, coumarins, flavonoids, alkaloids, and others.^45^ The *spotted knapweed*, an invasive species in the western U.S. prairies, inhibits the emergence and growth of native ground species through root secretions (catechins).^46^ *Stellera chamaejasme* L. and *Artemisia frigida* Willd. are common noxious weeds in the degraded grasslands of northern China. The former inhibits the growth of symbiotic plants mainly through the secretion of flavonoids by the root system,^47^ while the latter inhibits the germination of seeds and seedling growth of other plants through the release of volatile compounds (mainly terpenoids).^48^

In this study, 9 compounds were found to be the common up-regulated differential metabolites between R.EAE vs. R.BE, R.EAE vs. R.AE, SL.EAE vs SL.BE, and SL.EAE vs.SL.AE, including 2 aniline derivatives, 2 phenolic acids, 2 fatty acids, 1 aldehydes, 1 flavonoids and 1 lignansand, and were enriched in EAE. The contents of these compounds varied considerably between EAE and BE, EAE and AE. For example, in R.EAE, the N,N dimethylaniline level was 79.24 times higher than that of R.BE and 70.09 times that of R.AE; the caffeic acid level was 3.42 times higher than that of R.BE and 8.87 times that of R.AE; the 4- hydroxybenzaldehyde level was 3.42 times higher than that of R.BE and 8.87 times that of R.AE. Anilines significantly inhibit rice seed germination and root growth and also reduce DNA content in rice seedlings.^49^ At the same concentration (200 μg/ml), caffeic acid inhibited seed germination and seedling growth of *Arabidopsis thaliana* stronger than those of the commercial herbicides acetochlor and glyphosate.^50^ Caffeic acid and 4-hydroxybenzoic acid are considered to be allelochemicals of *Bidens pilosa* L., which contribute to the invasion of *Bidens pilosa* L.^51^ N,N-dimethylaniline,^52^ caffeic acid,^51,53,54^ 4-hydroxybenzoic acid,^51,53,54^ 4-hydroxybenzaldehyde,^54^ and cis-9-octadecenoic^55^ have been demonstrated to be allelochemicals, but eupatilin, 2-phenylethylamine, schisandragenol A, and 1-monolinolein have not yet been reported on their allelopathic activities. Therefore, N,N-dimethylaniline, caffeic acid, 4-hydroxybenzoic acid, 4-hydroxybenzaldehyde and cis-9-octadecenoic acid were potential allelochemicals in *L. sagitta*.

The present study confirmed the presence of allelopathy in *L. sagitt*a on the tested native species *P. pratensis*, *F. ovina*, *E. nutans*, *A. cristatum*, and significant inhibition of the growth and development of all these species. This suggests that the ability of *L. sagitta* to form a dominant species in degraded grassland is aided by its allelopathic mechanism, in addition to its own superb resistance.^56^ This result can explain the gradual replacement of dominance by *Gramineae* such as *P. pratensis* by noxious weed communities such as *L. sagitta* under continuous overgrazing pressure, and the subsequent loss of the former dominant species from the grassland. Such process forms a pattern dominated by noxious weeds, with a sparse mosaic distribution of *Gramineae* forages and short growth.^57,58^

*L*. *sagitta* currently exists in alpine grasslands in western Chinese grasslands as a invasive poisonous weeds, impacting on local biodiversity the development of grassland husbandry.^59^ Continuous mowing and replanting of grasses are effective restoration methods on grassland restoration.^4^ The continued presence of *L. sagitta* can impede the ability of native grassland vegetation to recover, and continuous mowing and biomass removal reduces competitive and undesirable species,^60^ therefore, in the restoration of grasslands degraded by *L. sagitta*, removing as much as possible of *L. sagitta* plants. Grassland restoration be accelerated by reseeding native species, and grassland ecosystem functioning and soil quality can be restored.^61^ However, replanting should be supplemented with forages that have strong resistance to it, such as *M. sativa*, while selection of *P. pratensis* and other *Gramineae* forages that are sensitive to it should be avoided. Local natural conditions and soil texture can also affect the establishment of native grasses. It is necessary to analyze the soil to avoid the residual effect of *L. sagitta* allelochemicals on the normal growth of plants.^62^ Further studies should look more closely at the allelochemicals of *L. sagitta*, their natural concentrations and the mechanism of inhibition.

## 5. Conclusions

This study adds to a large body of research showing that allelopathy is one of the mechanisms by which invasive plants dominance and that invasive plants can alter plant communities by inhibiting the growth and development of native species. The results of the study showed that allelochemicals were mainly present in the ethyl acetate extracts of stems-leaves and roots of *L. sagitta*. Based on this study, follow-up work will be carried out to isolate and purify the ethyl acetate extracts to find out the allelochemicals in *L. sagitta* and further investigate the specific mechanism of allelopathy.

However, this study had some limitations. For example, in the laboratory, climate, temperature, soil fauna, and soil microorganisms were excluded from interacting with and possibly neutralizing allelopathy. Therefore, allelopathy of *L. sagitta* should be further explored in future studies in natural conditions.

## Supplementary Material

Supplemental Material
