# Supplementary material for: Allelopathy and potential allelochemicals of Ligularia sagitta as an invasive plant
Source: Plant Signal Behav. 2024 Apr 28;19(1):2335025. doi: 10.1080/15592324.2024.2335025 (PMC11057658; doi:10.1080/15592324.2024.2335025)
Supplement: Supplemental Material [file KPSB_A_2335025_SM8607.pdf]

Supplementary Materials

Title:

Allelopathic effects of *Ligularia sagitta* extracts on forage seeds and seedlings

Authors:

Shengxiao Wang[1], Chenyue Wang[2], Jun Zhang[2], Kan Jiang[2 *] Fang Nian[1 *]

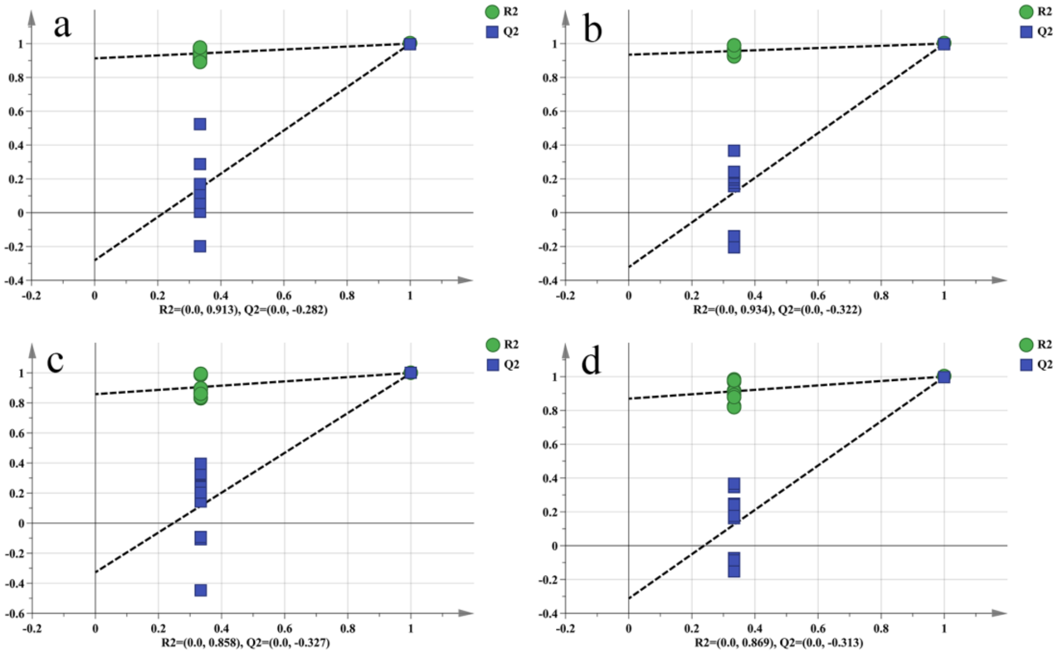

Supplementary Fig 1. Permutation test of OPLS-DA model. a: R.EAE vs. R.BE; b: R.EAE vs. R.AE; c: SL.EAE vs. SL.BE; d: SL.EAE vs. SL.AE. Roots ethyl acetate extract (R.EAE), Roots n-butanol extract (R.BE), Roots aqueous extract (R.AE), Stems-leaves ethyl acetate extract (SL.EAE), Stems-leaves n-butanol extract (SL.BE), Stems-leaves aqueous extract (SL.AE)

Supplementary Table 1 Effect of *L. sagitta* extract on the growth of *F. ovina* seedlings

Treatment solution		Inhibition（%）					
		Roots extracts			Stems-Leaves extracts		
		Shoots	Roots	Fresh weight	Shoots	Roots	Fresh weight
EAE (mg/mL)	0.25	9.48 ± 0.93 gh	3.65 ± 2.24 i	11.30 ± 1.40 fg	3.88 ± 2.63 e	7.18 ± 0.92 h	12.94 ± 2.82 def
	0.50	13.16 ± 0.59 fgh	10.63 ± 1.66 hi	26.78 ± 4.52 cd	6.26 ± 1.73 de	14.19 ± 2.56 g	19.54 ± 1.96 cd
	0.75	30.78 ± 2.67 cd	36.86 ± 4.83 cd	33.43 ± 4.16 bcd	18.02 ± 2.42 c	41.19 ± 2.56 cd	28.95 ± 3.69 bc
	1.00	41.40 ± 4.10 b	43.06 ± 2.40 c	37.39 ± 3.35 abc	35.29 ± 3.12 b	60.03 ± 2.64 b	39.23 ± 4.57 b
	2.00	57.49 ± 4.41 a	62.69 ± 3.20 b	46.97 ± 3.42 a	56.25 ± 3.73 a	87.15 ± 3.93 a	54.02 ± 4.23 a
BE (mg/mL)	0.25	6.74 ± 3.46 h	3.92 ± 3.17 i	5.99 ± 1.16 fg	3.21 ± 1.49 e	6.88 ± 1.19 h	5.09 ± 1.11 f
	0.50	10.71 ± 0.37 gh	4.50 ± 3.39 i	15.02 ± 2.10 ef	10.64 ± 0.98 cd	20.00 ± 2.85 f	5.38 ± 2.00 f
	0.75	14.18 ± 2.47 fgh	8.40 ± 4.21 hi	24.08 ± 3.58 de	11.88 ± 2.22 cd	27.78 ± 2.03 e	18.54 ± 3.63 cde
	1.00	16.39 ± 3.08 fg	16.26± 3.32 gh	31.12 ± 2.05 bcd	11.94 ± 1.04 cde	27.56 ± 2.57 e	21.38 ± 2.27 cd
	2.00	24.60 ± 1.75 de	31.49 ± 2.91 de	36.30 ± 4.05 bc	10.41 ± 2.01 cde	28.10 ± 3.40 e	25.91 ± 2.66 c
AE (mg/mL)	0.25	11.67 ± 2.37 gh	22.13 ± 3.06 fg	4.41 ± 2.84 g	11.28 ± 0.81 cde	6.64 ± 1.33 h	7.25 ± 2.40 ef
	0.50	20.42 ± 1.35 ef	23.76 ± 2.14 efg	24.61 ± 2.77 de	7.72 ± 3.93 de	21.13 ± 1.35 f	12.69 ± 1.58 def
	0.75	23.84 ± 3.43 de	26.75 ± 3.31 ef	24.87 ± 3.57 de	2.64 ± 0.41 e	30.61 ± 0.46 e	20.94 ± 1.33 cd
	1.00	26.91 ± 3.25 de	45.32 ± 1.46 c	27.54 ± 2.86 cd	6.23 ± 3.59 de	40.12 ± 4.11 d	22.98 ± 2.70 cd
	2.00	35.05 ± 1.33 bc	73.08 ± 4.01 a	39.59 ± 2.11 ab	8.70 ± 3.55 de	45.69 ± 3.39 b	23.13 ± 3.64 cd

Note: Values in the table are average value ± standard deviation (SD), lower case letters in the same column indicate significant differences between treatments at $p < 0.05$; Ethyl acetate extract (EAE), n-butanol extract (BE), aqueous extract (AE)

Supplementary Table 2. Effect of *L. sagitta* extract on the growth of *E. nutans* seedlings

Treatment solution		Inhibition（%）					
		Roots extracts			Stems-Leaves extracts		
		Shoots	Roots	Fresh weight	Shoots	Roots	Fresh weight
EAE (mg/mL)	0.25	7.73 ± 2.66 ef	6.60 ± 0.37 ghi	17.92 ± 1.35 d	2.86 ± 1.89 d	22.84 ± 3.09 d	35.50 ± 2.12 d
	0.50	17.43 ± 2.15 d	38.59 ± 3.46 d	25.60 ± 3.33 c	20.57 ± 1.77 c	51.22 ± 2.42 c	45.86 ± 4.93 c
	0.75	33.70 ± 1.34 c	56.41 ± 3.39 b	31.33 ± 2.63 c	22.25 ± 4.71 c	55.45 ± 3.89 c	53.10 ± 2.47 bc
	1.00	60.97 ± 2.34 b	61.63 ± 3.54 b	39.01 ± 2.79 b	44.88 ± 3.86 b	69.06 ± 3.01 b	59.28 ± 2.58 ab
	2.00	87.00 ± 0.42 a	87.98 ± 0.80 a	66.49 ± 1.81 a	70.19 ± 0.87 a	83.03 ± 0.50 a	66.73 ± 2.04 a
BE (mg/mL)	0.25	1.65 ± 1.75 g	6.55 ± 2.14 ghi	9.77 ± 3.63 ef	1.71 ± 0.85 d	2.25 ± 0.64 f	5.15 ± 2.77 h
	0.50	2.40 ± 3.05 g	4.46 ± 2.81 hi	3.53 ± 1.30 f	1.75 ± 0.64 d	4.21 ± 1.11 ef	20.94 ± 2.76 ef
	0.75	3.08 ± 1.82 g	12.06 ± 3.28 fg	4.29 ± 1.68 f	5.95 ± 1.07 d	3.87 ± 0.94 ef	17.46 ± 2.44 efg
	1.00	6.82 ± 0.61 f	33.28 ± 2.93 e	6.73 ± 1.39 f	7.75 ± 1.75 d	4.84 ± 1.47 ef	19.11 ± 3.63 ef
	2.00	10.70 ± 1.75 e	46.23 ± 3.09 c	16.42 ± 3.02 de	6.56 ± 1.39 d	21.85 ± 3.00 d	23.49 ± 1.60 e
AE (mg/mL)	0.25	2.11 ± 0.54 g	3.01 ± 1.79 i	5.59 ± 1.03 f	2.14 ± 0.844 d	1.89 ± 0.37 f	8.10 ± 1.35 h
	0.50	2.09 ± 0.90 g	5.60 ± 1.56 hi	6.45 ± 0.95 f	3.46 ± 0.86 d	3.42 ± 1.16 ef	9.11 ± 1.70 gh
	0.75	0.97 ± 0.10 g	9.64 ± 0.90 fgh	5.31 ± 1.10 f	3.27 ± 0.53 d	7.28 ± 1.77 ef	13.54 ± 2.20 fgh
	1.00	2.08 ± 0.62 g	15.07 ± 1.03 f	6.60 ± 1.48 f	1.61 ± 0.59 d	9.16 ± 1.28 e	13.50 ± 2.12 fgh
	2.00	2.01 ± 0.59 g	30.82 ± 3.81 e	7.31 ± 1.49 f	4.71 ± 1.58 d	17.46 ± 2.83 d	17.08 ± 3.04 efg

Note: Values in the table are average value ± standard deviation (SD), lower case letters in the same column indicate significant differences between treatments at $p < 0.05$; Ethyl acetate extract (EAE), n-butanol extract (BE), aqueous extract (AE)

Supplementary Table 3. Effect of *L. sagitta* extract on the growth of *A. cristatum* seedlings

Treatment solution		Inhibition（%）					
		Roots extracts			Stems-Leaves extracts		
		Shoots	Roots	Fresh weight	Shoots	Roots	Fresh weight
EAE (mg/mL)	0.25	19.72 ± 0.96 fg	30.09 ± 0.90 gh	13.22 ± 0.28 cd	13.26 ± 1.28 f	29.24 ± 3.46 g	10.13 ± 2.30 ef
	0.50	30.53 ± 0.35 d	54.92 ± 1.10 de	17.29 ± 1.85 c	28.42 ± 2.59 d	43.70 ± 3.33 ef	23.43 ± 1.84 d
	0.75	36.02 ± 3.23 c	62.75 ± 0.98 bcd	31.56 ± 2.38 b	34.90 ± 1.78 c	53.55 ± 2.40 cd	33.68 ± 4.21 c
	1.00	46.02 ± 0.92 b	71.09 ± 1.40 b	36.38 ± 2.27 b	43.91 ± 2.90 b	74.04 ± 3.41 b	42.27 ± 4.42 b
	2.00	66.22 ± 1.00 a	85.89 ± 0.92 a	48.39 ± 1.39 a	59.51 ± 2.33 a	88.43 ± 1.62 a	52.38 ± 3.60 a
BE (mg/mL)	0.25	19.22 ± 3.10 fg	13.13 ± 2.46 i	4.78 ± 1.16 d	4.40 ± 3.23 h	8.94 ± 2.82 i	4.44 ± 3.02 ef
	0.50	19.71 ± 1.68 fg	25.29 ±1.65 h	5.18 ± 2.92 d	7.07 ± 2.72 fg	33.34 ± 1.83 g	5.44 ± 2.60 ef
	0.75	21.93 ± 2.79 ef	36.41 ± 1.31 fg	5.81 ± 0.77 d	6.27 ± 3.49 fg	41.70 ± 2.82 f	12.96 ± 2.82 ef
	1.00	22.04 ± 3.71 ef	39.58 ± 0.74 f	7.83 ± 1.26 cd	8.75 ± 2.33 fg	49.40 ± 3.84 de	13.45 ± 1.53 ef
	2.00	23.39 ± 2.36 ef	51.66 ± 2.64 e	11.29 ± 2.67 cd	12.89 ± 1.99 e	51.88 ± 2.34 cd	13.00 ± 1.32 ef
AE (mg/mL)	0.25	15.12 ± 1.19 g	54.40 ± 4.17 de	7.32 ± 1.75 d	5.87 ± 1.53 gh	7.53 ± 0.75 i	3.32 ± 1.14 f
	0.50	21.87 ± 4.64 ef	56.18 ± 3.68 de	7.92 ± 1.13 cd	5.48 ± 3.97 gh	9.95 ± 1.60 i	5.16 ± 1.99 ef
	0.75	22.25 ± 1.51 ef	61.04 ± 4.28 cd	11.53 ± 2.80 cd	6.41 ± 0.73 fg	11.47 ± 2.85 i	5.29 ± 1.21 ef
	1.00	24.44 ± 1.79 ef	65.32 ± 1.14 bc	13.13 ± 2.94 cd	7.11 ± 1.83 fg	22.80 ± 1.67 h	10.97 ± 1.60 ef
	2.00	26.53 ± 2.69 de	69.87 ± 1.35 b	13.95 ± 1.76 cd	8.79 ± 0.66 fg	56.31 ± 3.70 c	12.65 ± 2.63 e

Note: Values in the table are average value ± standard deviation (SD), lower case letters in the same column indicate significant differences between treatments at $p < 0.05$; Ethyl acetate extract (EAE), n-butanol extract (BE), aqueous extract (AE)

Supplementary Table 4. Effect of *L. sagitta* extract on the growth of *M. sativa* seedlings

Treatment solution		Inhibition（%）					
		Roots extracts			Stems-Leaves extracts		
		Shoots	Roots	Fresh weight	Shoots	Roots	Fresh weight
EAE (mg/mL)	0.25	3.59 ± 1.28 e	12.34 ± 0.85 i	4.08 ± 2.73 b	14.58 ± 2.00 f	4.72 ± 1.00 g	4.68 ± 4.37 e
	0.50	13.81 ± 1.45 cd	25.23 ± 2.09 h	7.68 ± 1.63 b	16.11 ± 0.26 f	8.94 ± 1.67 fg	4.41 ± 2.03 e
	0.75	16.98 ± 3.95 cd	31.70 ± 1.55 g	9.38 ± 2.84 b	19.09 ± 2.92 ef	14.26 ± 2.59 ef	14.59 ± 2.52 bcd
	1.00	22.77 ± 3.70 bc	34.69 ± 2.88 fg	16.83 ± 2.76 b	23.98 ± 0.32 cde	19.83 ± 4.83 de	17.85 ± 3.49 b
	2.00	39.18 ± 2.70 a	38.73 ± 3.19 f	46.37 ± 3.94 a	33.05 ± 2.30 ab	58.07 ± 2.80 a	30.50 ± 1.68 a
BE (mg/mL)	0.25	15.37 ± 2.07 cd	29.98 ± 3.07 g	8.84 ± 2.87 b	14.71 ± 2.15 f	7.12 ± 1.63 fg	4.48 ± 1.84 e
	0.50	18.71 ± 3.63 cd	45.97 ± 3.03 e	13.16 ± 3.37 b	20.59 ± 3.38 ef	12.03 ± 1.45 fg	3.21 ± 0.48 e
	0.75	22.58 ± 2.69 bc	51.82 ± 3.95 d	14.78 ± 3.90 b	22.70 ± 2.54 de	21.00 ± 3.92 de	7.51 ± 2.73 de
	1.00	24.65 ± 0.23 bc	57.21 ± 1.57 c	18.84 ± 4.45 ab	27.05 ± 1.61 bcd	20.93 ± 0.33 de	8.20 ± 2.08 de
	2.00	26.11 ± 2.19 bc	60.26 ± 1.59 c	19.63 ± 3.36 ab	29.72 ± 2.55 bc	35.27 ± 1.29 c	16.09 ± 1.92 bc
AE	0.25	10.48 ± 1.55 de	55.92 ± 3.24 cd	16.19 ± 2.97 b	23.69 ± 1.78 cde	23.23 ± 2.71 d	8.45 ± 2.36 de
	0.50	18.04 ± 1.99 cd	68.27 ± 2.22 b	27.47 ± 2.46 ab	28.45 ± 3.34 bcd	37.03 ± 2.81 c	8.35 ± 1.57 e

(mg/mL)	0.75	25.08 ± 2.41 bc	72.52 ± 2.64 b	31.58 ± 0.27 ab	31.38 ± 1.71 ab	46.10 ± 1.55 b	9.01 ± 2.09 cde
	1.00	31.27 ± 4.17 ab	79.72 ± 0.14 a	35.67 ± 3.10 ab	36.01 ± 1.93 a	51.11 ± 4.37 b	14.77 ± 2.04 bcd
	2.00	33.19 ± 2.03 ab	83.96 ± 1.18 a	36.16 ± 2.17 ab	35.97 ± 1.46 a	62.98 ± 5.54 a	33.10 ± 2.92 a

Note: Values in the table are average value ± standard deviation (SD), lower case letters in the same column indicate significant differences between treatments at $p < 0.05$; Ethyl acetate extract (EAE), n-butanol extract (BE), aqueous extract (AE)

Supplementary Table 5 Statistics of differential metabolites in different extracts of *L. sagitta*

Groups	Type	Number	Main categories and percentages
R.EAE vs R.BE	up	19	terpenoids (21.05%) lipids (21.05%) alkaloids (15.79%)
	down	12	organic acids (33.33%)
R.EAE vs R.AE	up	21	terpenoids (19.05%) lipids (19.05%)<br>organic acids (19.05%)
	down	43	organic acids (30.23%) carbohydrates (18.60%)<br>amino acids and their derivatives (18.60%)
SLEAE vs SL.BE	up	31	terpenoids (19.35%) organic acids (19.35%)<br>flavonoids (16.13%)
	down	50	organic acids (18.0%) lipids (16.0%)<br>amino acids and their derivatives (16.0%)
SL.EAE vs SL.AE	up	27	organic acids (25.93%) flavonoids (18.52%)<br>terpenoids (14.81%)
	down	39	organic acids (28.21%)<br>amino acids and their derivatives (25.64%)

Supplementary Table 6. Summary of differential metabolites in R.EAE vs. R.BE

NO.	Compounds	m/z	Type	VIP-value	*p*- value	Fold Change
1	2-Isopropylaniline	136.1123	up	2.37	6.18E-05	143.9780
2	2-Phenylethylamine	122.0967	up	4.75	1.29E-07	93.7534
3	N,N-Dimethylaniline	122.0965	up	10.67	1.12E-05	79.2441
4	Schizandrol A	477.2130	up	2.54	6.24E-07	12.7639
5	1-Monolinolein	355.2856	up	1.95	4.51E-07	10.4686
6	Arachidonic acid	305.2475	up	1.59	3.83E-04	8.9150
7	Embelin	277.1800	up	3.69	2.98E-06	8.2657
8	Anise oil	235.1329	up	3.05	1.25E-04	7.5646
9	Estradiol-17β-glucuronide	448.5163	up	3.02	8.83E-05	6.2755
10	Oridonin	363.1816	up	2.25	1.77E-04	6.2323
11	4-Hydroxybenzoic acid	137.0249	up	3.89	6.70E-04	5.7279
12	Eupatilin	343.0828	up	3.29	1.03E-08	5.5097
13	4-Hydroxybenzaldehyde	123.0446	up	3.06	3.00E-07	5.1421
14	4-Ethylphenol	245.1536	up	2.44	3.39E-04	4.8906
15	Pterosin B	217.1236	up	1.84	2.51E-03	4.5071
16	4-Phenyl-3-buten-2-one	147.0806	up	1.52	2.34E-05	3.5579
17	cis-9-Octadecenoic	281.2480	up	2.66	6.18E-07	3.5418
18	Caffeic acid	179.0352	up	6.18	8.42E-06	3.4154
19	Gibberellin A4	331.1554	up	2.42	1.20E-05	2.5636
20	1,3-bis(4-methoxybenzyl)-2-phenylhexahydropyrimidine	403.2344	down	3.16	8.60E-05	0.0498
21	DL-Norvaline	118.0864	down	1.91	7.62E-06	0.0415
22	Quinone	126.0550	down	3.06	1.85E-05	0.0413
23	Gluconic acid	195.0512	down	1.38	4.15E-05	0.0394
24	Adenosine	268.1042	down	1.67	3.14E-04	0.0372
25	methyl 3,4,5-trihydroxycyclohex-1-ene-1-carboxylate	171.0653	down	1.63	1.19E-04	0.0324
26	Citric acid	191.0200	down	1.54	1.97E-03	0.0294
27	2-Hydroxycinnamic acid	182.0813	down	2.50	2.15E-04	0.0275
28	4-Guanidinobutyric acid	146.0925	down	2.51	1.17E-05	0.0257
29	L-Norleucine	132.1021	down	2.60	1.36E-04	0.0204
30	N-Acetylhistamine	154.0976	down	6.23	2.63E-08	0.0178
31	8-Aminooctanoic acid	160.1334	down	1.67	8.46E-05	0.0079

Supplementary Table 7. Summary of differential metabolites in R.EAE vs. R.AE

NO.	Compounds	m/z	Type	VIP-value	*p*- value	Fold Change
1	Eupatilin	343.0828	up	3.10	2.16E-09	376.0700
2	2-Isopropylaniline	136.1123	up	2.02	6.16E-05	152.7570
3	2-Phenylethylamine	122.0967	up	4.06	1.29E-07	92.2602
4	N,N-Dimethylaniline	122.0965	up	9.10	1.13E-05	70.0886
5	Embelin	277.1800	up	3.34	1.86E-06	65.9545
6	Schizandrol A	477.2130	up	2.24	4.10E-07	49.4591
7	4-Hydroxybenzoic acid	137.0249	up	3.64	3.53E-08	44.4500
8	Estradiol-17β-glucuronide	448.5163	up	2.77	4.89E-05	39.9634
9	Oridonin	363.1816	up	2.05	1.02E-04	27.4262
10	1-Monolinolein	355.2856	up	1.72	3.02E-07	26.1988
11	4-Hydroxybenzaldehyde	123.0446	up	2.83	1.62E-07	18.2867
12	4-Ethylphenol	245.1536	up	2.25	1.80E-04	14.4774
13	cis-9-Octadecenoic	281.2480	up	2.59	1.66E-07	14.2696
14	5-hydroxy-4-methoxy-5,6-dihydro-2H-pyran-2-one	145.0496	up	1.97	4.79E-02	14.1865
15	Pterosin B	217.1236	up	1.68	1.39E-03	9.4130
16	Anise oil	235.1329	up	2.64	7.14E-05	9.3129

17	Caffeic acid	179.0352	up	5.91	2.72E-06	8.8740
18	Gibberellin A4	331.1554	up	2.39	3.44E-06	5.3940
19	PC O-18:2	520.3403	up	1.73	5.88E-05	5.3634
20	3,4-Dimethylbenzoic acid	151.1195	up	1.53	1.71E-04	5.0265
21	3-Methylcrotonylglycine	158.0814	up	1.94	2.86E-05	3.0994
22	2-Oxobutyric acid	101.0246	down	1.61	6.25E-04	0.4990
23	Quercetin 3-alpha-L-arabinofuranoside (Avicularin)	433.0773	down	3.10	4.03E-05	0.4889
24	Succinic acid	117.0195	down	4.61	1.63E-06	0.4057
25	D-(-)-Fructose	179.0563	down	1.65	4.18E-05	0.3090
26	7-Hydroxycoumarine	163.0390	down	2.70	3.40E-05	0.2894
27	D-(+)-Arabitol	151.0614	down	1.60	1.98E-04	0.2507
28	2-Isopropylmalic acid	175.0614	down	1.78	4.30E-05	0.2067
29	α,α-Trehalose	341.1090	down	2.85	3.15E-04	0.2011
30	Pipecolic acid	130.0864	down	4.13	2.41E-05	0.1970
31	D-(+)-Mannose	179.0564	down	4.06	1.32E-05	0.1460
32	4-Oxoproline	128.0355	down	2.85	7.75E-06	0.1393
33	D-Proline	116.0708	down	3.70	1.78E-06	0.1316
34	Methyl-beta-galactopyranoside	239.0774	down	2.09	1.67E-07	0.1313
35	Purine	119.0352	down	2.25	6.15E-06	0.1238
36	D-Xylonic acid	165.0407	down	1.54	1.52E-05	0.1118
37	3-Hydroxy-3-Methylpentane-1,5-Dioic Acid	163.0602	down	2.11	1.55E-04	0.1117
38	3-Hydroxy-3-methylglutaric acid	161.0458	down	3.16	2.65E-05	0.1007
39	D-Ribono-1,4-lactone	149.1285	down	1.90	2.06E-03	0.0878
40	4-Acetamidobutyric Acid	128.0707	down	3.03	4.99E-02	0.0872
41	D-(+)-Proline	116.0707	down	4.28	7.70E-06	0.0824
42	Hexadecanamide	256.2635	down	3.02	2.20E-04	0.0745
43	D-(-)-Quinic acid	191.0564	down	2.04	2.35E-05	0.0739
44	Stearamide	284.2948	down	2.17	2.02E-05	0.0722
45	1,3-bis(4-methoxybenzyl)-2-phenylhexahydropyrimidine	403.2344	down	2.42	1.41E-06	0.0613
46	N-Benzylformamide	136.0759	down	1.63	1.18E-05	0.0388
47	(2R,3S,4S,5R,6S)-2-(hydroxymethyl)-6-phenoxyoxane-3,4,5-triol	274.1285	down	2.96	6.86E-06	0.0332
48	1,3-dimethyl-1H-thieno[2,3-c]pyrazole-5-carbohydrazide	209.0491	down	2.72	5.75E-08	0.0314
49	methyl 2-[(2-acetyl-3-oxo-1-butenyl)amino]acetate	200.0918	down	2.00	3.50E-05	0.0309
50	DL-Malic acid	133.0144	down	5.77	1.75E-08	0.0306
51	DL-Norvaline	118.0864	down	2.01	9.24E-04	0.0274
52	Anisic aldehyde	154.0864	down	2.02	9.37E-08	0.0255
53	Dopamine	154.0863	down	2.02	6.13E-08	0.0254
54	D-(+)-Malic acid	133.0145	down	1.69	7.14E-07	0.0238
55	5-Aminovaleric acid	116.0719	down	1.73	2.14E-08	0.0225
56	Propylparaben	198.1127	down	1.70	1.32E-02	0.0185
57	Gluconic acid	195.0512	down	1.78	7.03E-06	0.0176
58	L-Norleucine	132.1021	down	2.63	7.17E-07	0.0148
59	2-Hydroxycinnamic acid	182.0813	down	3.04	1.01E-04	0.0139
60	D-(+)-Pyroglutamic Acid	130.0501	down	2.17	3.29E-06	0.0139
61	N-Acetylhistamine	154.0976	down	7.61	3.83E-05	0.0088
62	4-Guanidinobutyric acid	146.0925	down	3.92	4.23E-07	0.0079
63	N-[(-)-Jasmonoyl]-(L)-Isoleucine	344.1820	down	2.15	2.20E-06	0.0039
64	Citric acid	191.0200	down	3.92	4.75E-04	0.0035

Supplementary Table 8. Summary of differential metabolites in SL.EAE vs. SL.BE

NO.	Compounds	m/z	Type	VIP-value	*p*- value	Fold Change
1	D-Limonene	154.1590	up	3.12	1.98E-09	113.9990
2	Eupatilin	343.0828	up	2.26	6.63E-07	28.6820
3	Schizandrol A	477.2130	up	1.95	1.18E-05	18.4531
4	4-Hydroxy-2,5-dimethyl-3(2H)furanone	127.0404	up	3.66	4.39E-07	17.1482
5	Isoferulic acid	193.0509	up	2.28	3.00E-07	15.1373
6	Dihydrokawain	233.1172	up	3.90	1.68E-05	12.6032
7	beta-Caryophyllene	205.1952	up	2.13	9.68E-04	12.2630
8	1-Monolinolein	355.2856	up	2.51	5.95E-05	11.1085
9	Sequoyitol	195.0866	up	1.76	7.45E-05	10.2673
10	Olivetol	181.1224	up	1.69	2.90E-04	10.0362
11	2,4-Dimethylbenzaldehyde	135.0806	up	1.84	5.49E-05	9.3176
12	cis-9-Octadecenoic	281.2480	up	1.87	6.28E-07	9.2869
13	N,N-Dimethylaniline	122.0965	up	6.44	6.17E-04	9.0564
14	Guan-fu base A	430.2229	up	2.49	2.66E-05	8.7854
15	Picrotin	309.0981	up	1.61	1.09E-06	8.3403
16	4,7-dimethoxy-1H-phenalen-1-one	258.1126	up	1.86	4.82E-06	8.2548
17	2-Phenylethylamine	122.0967	up	2.18	6.50E-04	8.1094
18	Corchorifatty acid F	327.2176	up	1.64	7.56E-06	7.6773
19	Caffeic acid	179.0352	up	3.43	2.22E-03	7.4784
20	1,3-Diphenylacetone	228.1384	up	4.62	4.81E-08	7.1320
21	Crocetin	329.1749	up	1.73	1.37E-04	5.9748
22	Quercetin	301.0355	up	1.91	5.49E-05	5.9450
23	4-Hydroxybenzaldehyde	123.0446	up	2.14	9.19E-06	5.1386
24	β-Lapachone	243.1017	up	1.56	4.43E-04	3.8479
25	(-)-Caryophyllene oxide	203.1796	up	1.57	3.29E-03	3.4740
26	3-Methylcrotonylglycine	158.0814	up	2.73	8.53E-03	2.7104
27	Quercetin-3-D-glucoside	465.1028	up	2.04	2.73E-04	2.6326
28	4-Hydroxybenzoic acid	137.0249	up	2.82	2.31E-04	2.6046
29	Sinomenine	330.1702	up	1.60	3.97E-05	2.3250
30	Kaempferol	287.0551	up	1.88	2.29E-03	2.3013
31	Pinoresinol dimethyl ether	387.1806	up	1.82	5.82E-04	2.2030
32	D-(-)-Fructose	179.0563	down	1.63	1.81E-02	0.4891
33	Chlorogenic acid	353.0878	down	4.14	3.84E-07	0.4368
34	Succinic acid	117.0195	down	3.80	1.09E-04	0.4359
35	Hexadecanamide	256.2635	down	2.27	1.21E-02	0.3550
36	α-Lactose	387.1144	down	1.65	2.86E-02	0.3238
37	Oleamide	282.2791	down	4.84	4.60E-03	0.3234
38	D-(+)-Proline	116.0707	down	2.89	2.12E-03	0.3121
39	LPA 18:2	433.2356	down	1.90	2.51E-03	0.2805
40	4-Oxoproline	128.0355	down	3.21	1.35E-02	0.2516
41	4-Acetamidobutyric Acid	128.0707	down	1.84	6.27E-07	0.2359
42	Nicotinic acid	124.0395	down	1.51	2.35E-04	0.2112
43	Esculin	339.0721	down	2.46	2.44E-04	0.1981
44	Nicotinamide	123.0554	down	2.50	9.42E-04	0.1893
45	α,α-Trehalose	341.1090	down	1.83	5.22E-04	0.1848
46	Uracil	113.0347	down	1.68	3.26E-04	0.1730
47	Stearamide	284.2948	down	1.96	3.49E-05	0.1627
48	Guvacoline	142.0865	down	1.81	1.61E-04	0.1611
49	7-Hydroxycoumarine	163.0390	down	2.77	3.67E-05	0.1602
50	Azetidine-2-carboxylic acid	102.0551	down	1.53	6.62E-05	0.1573
51	Ecliptasaponin A	633.4010	down	1.75	4.06E-05	0.1441
52	D-Proline	116.0708	down	6.40	2.27E-04	0.1318
53	2-Hydroxycinnamic acid	182.0813	down	2.23	8.06E-04	0.1293
54	1-allyl-9,9,9a-trimethyl-9,9a-dihydro-1H-imidazo[1,2-a]indol-2(3H)-one	257.1650	down	1.61	7.04E-06	0.1208

55	Pyridoxine	170.0813	down	2.98	2.22E-05	0.1104
56	Pomolic acid beta-D-glucopyranosyl ester	633.4014	down	1.80	7.96E-07	0.1095
57	Robinetin	303.0501	down	2.31	1.11E-05	0.1043
58	DL-Stachydrine	144.1020	down	1.90	1.04E-05	0.1035
59	D-(-)-Quinic acid	191.0564	down	2.91	5.90E-07	0.0985
60	Uridine	243.0626	down	1.77	1.54E-05	0.0962
61	Ecgonine methyl ester	200.1282	down	1.60	1.60E-04	0.0933
62	Quinone	126.0550	down	2.24	5.01E-04	0.0879
63	PC O-18:2	520.3403	down	2.00	1.63E-04	0.0851
64	DL-Malic acid	133.0144	down	2.29	4.29E-04	0.0846
65	gamma-caprolactone	132.1021	down	1.56	3.27E-02	0.0836
66	PC O-18:3	518.3254	down	1.74	2.74E-06	0.0806
67	Adenine	136.0620	down	1.96	2.26E-04	0.0781
68	Erythrono-1,4-lactone	136.0620	down	1.96	2.26E-04	0.0781
69	Kaji-ichigoside F1	649.3961	down	2.83	7.65E-07	0.0711
70	PC O-16:0	496.3407	down	1.86	1.04E-04	0.0684
71	Choline	104.1071	down	1.79	1.33E-02	0.0652
72	Indole-3-acrylic acid	188.0707	down	1.93	3.77E-06	0.0595
73	Adenosine	268.1042	down	3.48	7.67E-04	0.0527
74	5-Aminovaleric acid	116.0719	down	1.95	4.27E-06	0.0398
75	N-Acetylhistamine	154.0976	down	6.29	1.27E-04	0.0365
76	DL-Norvaline	118.0864	down	3.58	6.97E-03	0.0350
77	L-Phenylalanine	164.0720	down	1.54	5.00E-08	0.0338
78	isoleucine	132.1021	down	1.69	4.82E-05	0.0287
79	L-Norleucine	132.1021	down	5.16	4.68E-05	0.0260
80	(2R,3S,4S,5R,6S)-2-(hydroxymethyl)-6-phenoxyoxane-3,4,5-triol	274.1285	down	1.78	3.41E-05	0.0253
81	4-Guanidinobutyric acid	146.0925	down	1.61	7.99E-04	0.0133

Supplementary Table 9. Summary of differential metabolites in SL.EAE vs. SL.AE

NO.	Compounds	m/z	Type	VIP-value	*p*- value	Fold Change
1	Eupatilin	343.0828	up	1.88	5.77E-07	649.4590
2	beta-Caryophyllene	205.1952	up	1.81	7.05E-04	345.6320
3	Schizandrol A	477.2130	up	1.63	9.30E-06	117.2020
4	1-Monolinolein	355.2856	up	2.14	4.24E-05	105.7370
5	Sequoyitol	195.0866	up	1.51	5.16E-05	100.3560
6	4-Hydroxy-2,5-dimethyl-3(2H)furanone	127.0404	up	3.06	5.47E-08	93.5793
7	1,3-Diphenylacetone	228.1384	up	4.04	1.25E-08	82.6283
8	Sarpogrelate	430.2232	up	2.54	2.22E-08	58.6512
9	N,N-Dimethylaniline	122.0965	up	5.52	4.22E-04	47.0162
10	4,7-dimethoxy-1H-phenalen-1-one	258.1126	up	1.60	1.39E-07	46.7777
11	Guan-fu base A	430.2229	up	2.13	1.89E-05	39.4287
12	Crocetin	329.1749	up	1.53	6.55E-05	38.9304
13	2-Phenylethylamine	122.0967	up	1.88	4.32E-04	34.9384
14	Dihydrokawain	233.1172	up	3.26	1.37E-05	30.5823
15	2,4-Dimethylbenzaldehyde	135.0806	up	1.56	4.05E-05	25.5749
16	Kaempferol	287.0551	up	2.01	2.00E-04	24.5812
17	Gibberellin A7	329.1396	up	1.62	1.13E-05	24.0735
18	4-Hydroxybenzoic acid	137.0249	up	2.86	2.81E-05	20.0296
19	4-Hydroxybenzaldehyde	123.0446	up	1.89	4.12E-06	16.7868
20	cis-9-Octadecenoic	281.2480	up	1.57	5.32E-07	15.7812
21	Quercetin	301.0355	up	1.65	3.87E-05	15.1390
22	Quercetin-3-D-glucoside	465.1028	up	2.04	1.99E-05	14.6748
23	Isoferulic acid	193.0509	up	1.85	1.13E-07	12.4955
24	Pinoresinol dimethyl ether	387.1806	up	1.89	3.95E-04	7.9152

25	3-Methylcrotonylglycine	158.0814	up	2.58	2.31E-03	6.1088
26	Abscisic Acid	247.1330	up	2.14	7.85E-04	4.9004
27	Caffeic acid	179.0352	up	2.52	1.54E-03	3.3640
28	Levulinic acid	115.0402	down	4.29	1.42E-04	0.4322
29	Chlorogenic acid	353.0878	down	3.58	4.26E-07	0.4089
30	Quercetin 3-alpha-L-arabinofuranoside (Avicularin)	433.0773	down	2.03	1.36E-02	0.3843
31	Hexadecanamide	256.2635	down	1.88	6.41E-04	0.3522
32	Oleamide	282.2791	down	4.63	6.89E-05	0.2584
33	Dulcitol	181.0720	down	1.61	1.88E-02	0.2061
34	α-Lactose	387.1144	down	1.88	8.30E-04	0.1965
35	3-Hydroxy-3-methylglutaric acid	161.0458	down	3.72	1.60E-05	0.1658
36	D-Proline	116.0708	down	4.64	2.40E-03	0.1607
37	Pyridoxine	170.0813	down	1.97	6.34E-06	0.1584
38	Stearamide	284.2948	down	1.73	1.52E-04	0.1432
39	α,α-Trehalose	341.1090	down	2.01	1.08E-04	0.1115
40	Guvacoline	142.0865	down	1.83	1.67E-06	0.1107
41	Nicotinic acid	124.0395	down	1.85	1.01E-04	0.1064
42	4-Oxoproline	128.0355	down	4.53	2.53E-05	0.1059
43	Succinic acid	117.0195	down	8.14	8.19E-07	0.1010
44	Pipecolic acid	130.0864	down	3.09	5.05E-05	0.0941
45	7-Hydroxycoumarine	163.0390	down	3.22	1.19E-06	0.0867
46	2-Hydroxycinnamic acid	182.0813	down	2.47	8.52E-04	0.0736
47	2-Isopropylmalic acid	175.0614	down	2.57	9.74E-07	0.0690
48	Adenosine	268.1042	down	2.59	7.51E-06	0.0629
49	D-(+)-Proline	116.0707	down	6.26	1.79E-04	0.0600
50	Ecgonine methyl ester	200.1282	down	1.69	1.35E-05	0.0580
51	D-Ribono-1,4-lactone	149.1285	down	1.71	1.29E-06	0.0549
52	Choline	104.1071	down	1.78	9.45E-07	0.0455
53	N-Acetylhistamine	154.0976	down	4.67	8.67E-07	0.0438
54	N-Acetyl-L-leucine	174.1126	down	1.77	1.17E-05	0.0424
55	DL-Stachydrine	144.1020	down	2.72	6.38E-07	0.0364
56	Ecgonine	186.1126	down	1.84	3.26E-03	0.0307
57	L-Threonic acid-1,4-lactone	119.0341	down	2.62	1.89E-06	0.0289
58	D-(-)-Quinic acid	191.0564	down	4.58	4.84E-08	0.0285
59	Dl-3-Hydroxynorvaline	114.0563	down	2.45	1.66E-03	0.0206
60	5-Aminovaleric acid	116.0719	down	2.36	5.04E-06	0.0184
61	DL-Norvaline	118.0864	down	4.21	2.27E-04	0.0181
62	DL-Malic acid	133.0144	down	4.68	4.20E-07	0.0146
63	L-Norleucine	132.1021	down	6.46	3.23E-05	0.0112
64	D-(+)-Pyroglutamic Acid	130.0501	down	2.90	3.70E-05	0.0095
65	Citric acid	191.0200	down	3.18	1.65E-07	0.0067
66	4-Guanidinobutyric acid	146.0925	down	2.21	1.54E-06	0.0048
